# Prospective Observational Study Evaluating Systemic Hormones and Corneal Crosslinking Effects in Keratoconus

**DOI:** 10.1016/j.xops.2023.100364

**Published:** 2023-07-11

**Authors:** Lyly Van, Sashia Bennett, Sarah E. Nicholas, Jesper Hjortdal, Tina B. McKay, Dimitrios Karamichos

**Affiliations:** 1 Department of Ophthalmology, Dean McGee Eye Institute, University of Oklahoma Health Sciences Center, Oklahoma City, Oklahoma; 2 Department of Ophthalmology, Aarhus University Hospital, 8200 Aarhus N, Denmark; 3 North Texas Eye Research Institute, University of North Texas Health Science Center, Fort Worth, Texas; 4 Department of Pharmaceutical Sciences, University of North Texas Health Science Center, Fort Worth, Texas; 5 Department of Anesthesia, Critical Care and Pain Medicine, Massachusetts General Hospital, Harvard Medical School, Boston, Massachusetts; 6 Department of Pharmacology and Neuroscience, University of North Texas Health Science Center, Fort Worth, Texas

**Keywords:** Cornea, Corneal crosslinking, Estrogen, Keratoconus, Hormones

## Abstract

**Purpose:**

To evaluate associations between hormone levels and corneal parameters in patients with keratoconus (KC), before and after photooxidative corneal collagen crosslinking (CXL).

**Design:**

Prospective, observational cohort study.

**Participants:**

Twenty-eight patients with KC who were scheduled for CXL at Aarhus University Hospital in Denmark.

**Methods:**

Androgen (dehydroepiandrosterone sulfate [DHEA-S]) and estrogen (estrone and estriol) plasma levels were measured and clinical assessments were performed before CXL and 2 to 3 months post-CXL, comparing the CXL eye with the control eye from the same participant.

**Main Outcome Measures:**

Associations between hormone levels and maximum corneal curvature (K_max_) and minimum central corneal thickness (CCt_min_) before and after CXL.

**Results:**

Corneal collagen crosslinking was associated with a 2% reduction in K_max_ values in the CXL eye, post-CXL, from baseline (median, 56.8 diopters [D]; 95% confidence interval [CI], 50.4–60.3) to the second visit (55.7 D; 95% CI, 50.4–58.8; *P* < 0.001). Systemic DHEA-S levels were 5 to 6 orders of magnitude higher than estriol or estrone concentrations in plasma. Importantly, estriol levels, rather than DHEA-S or estrone levels, were more closely correlated with K_max_ before CXL (Spearman’s *r* = 0.55, *P* = 0.01). Post-CXL K_max_ and CCt_min_ were not associated with DHEA-S, estrone, or estriol plasma levels at the same timepoint.

**Conclusions:**

This study provides supporting evidence based on a KC clinical population that systemic estrogen levels may influence corneal parameters (curvature and thickness) pre-CXL. Further studies evaluating the interplay between the therapeutic benefits of CXL and systemic hormone distributions are needed to determine if perturbation of the local corneal microenvironment influences endocrine function.

**Financial Disclosure(s):**

The authors have no proprietary or commercial interest in any materials discussed in this article.

Keratoconus (KC) is a common corneal dystrophy that affects 1:500 to 1:2000 people worldwide.^1^^,^^2^ Current treatment modalities focus on correcting refractive error via specialized contact lenses or intrastromal segments, while corneal transplantation is required for severe cases, such as those associated with corneal scarring or formation of hydrops.^3^ Photooxidative corneal collagen crosslinking (CXL) with riboflavin was first reported to be efficacious in reducing KC severity in a clinical population in 2003^4^ and has since gained prominence as a safe, Food and Drug Administration-approved treatment designed to reduce corneal thinning and progression.^5^, ^6^, ^7^ Corneal collagen crosslinking involves a chemical process induced by exposure of the cornea to riboflavin and ultraviolet light, which leads to the formation of reactive oxygen species that react with carbonyl groups on amino acid side chains.^7^ This reaction promotes the formation of chemical crosslinks within collagen fibrils and between surrounding proteoglycans that are highly abundant within the stroma.^8^ Physiologically crosslinking by lysyl oxidase or age-related glycation end products during prolonged hyperglycemia are slow reactions by comparison that likewise influence the biomechanical properties of the cornea and have been inversely associated with the development of KC.^9^

In terms of the etiology underlying KC, studies have suggested that genetic factors,^10^ eye rubbing,^11^ mitochondrial dysfunction,^12^ increased inflammation,^13^ and elevated oxidative stress^14^ may be contributing factors. Although the pathophysiology of KC remains largely unclear, growing evidence suggests that differential distribution of systemic hormones may be associated with KC onset.^15^^,^^16^ In a case-control study, we detected higher dehydroepiandrosterone sulfate (DHEA-S) and lower estrone salivary levels in patients with KC compared with age- and sex-matched controls, which correlated with elevated proinflammatory factors, suggesting that differential systemic hormone levels may be a key driver of the structural changes in the cornea.^17^ These findings are consistent with recent reports by other groups identifying connections between KC and systemic hormones.^16^^,^^18^ Retrospective analyses have reported advanced corneal thinning in patients with KC during pregnancy^19^^,^^20^ and after hormone replacement therapy^21^ and in vitro fertilization,^22^ in previously stable KC cases, providing retrospective anecdotal evidence that altered estrogen levels may promote KC progression.

In this article, we performed a prospective observational study of 28 adults with KC scheduled for CXL and evaluated clinical characteristics in response to CXL, comparing the naive CXL-treated eye with the other eye from the same subject (control). We hypothesized that baseline hormone levels may influence the effectiveness of CXL in terms of corneal curvature and central corneal thickness. To address this hypothesis, we measured DHEA-S, estrone, and estriol plasma levels in patients with KC before and after CXL in concert with clinical assessments. This study provides supporting evidence for a potential role for systemic hormones in influencing corneal structure in the context of KC and investigates whether corneal parameters post-CXL are associated with circulating DHEA-S and estrogen levels.

## Methods

### Study Design

A prospective, observational study was performed on KC subjects scheduled for CXL at Aarhus University Hospital, Denmark. The research protocol was approved by The Central Denmark Region Committees on Health Research Ethics (protocol number: 1-10-72-127- 16). Informed consent was obtained from all subjects before enrollment and in accordance to the tenets of the Declarations of Helsinki. Inclusion criteria for enrollment included documented KC diagnosis and progression defined as an increase in K_max_ of ≥ 1.5 diopter (D) over a 3- to 6-month period. The exclusion criteria were the presence of other eye diseases, as well as previous CXL or other types of corneal surgery in the experimental eye. Plasma samples were collected before CXL and at the second visit ≥ 2 to 3 months after CXL and stored at −80° C until further analysis. Corneal parameters, such as maximum corneal curvature (K_max_), minimum central corneal thickness (CCt_min_), and topographic KC classification, demographic information, such as age and sex, and prior ocular treatments, including CXL or corneal transplantation, were recorded. Two samples were previously included in a preliminary study.^15^

### Hormone Analysis

Plasma samples were analyzed according to the following manufacturer’s protocol: DHEA-S (MBS3803557; MyBioSource), estrone (MBS3803033; MyBioSource), and estriol (MBS3803557; MyBioSource). Briefly, standards and unknowns were thawed and incubated with the coated antibody plates and conjugated antibody for 1 hour at 37° C, followed by washing and incubation with the chromogen solution for 15 minutes at room temperature and then addition of a stop solution. Absorbance measurements at 450 nm were measured using a spectrophotometer, and the unknown concentrations were interpolated from a standard curve generated using pure compounds of known concentration.

### Statistical Analysis

Statistical analyses were performed using GraphPad Prism (GraphPad Software). Normality was assessed using a Kolmogorov-Smirnov normality test. Correlations were evaluated using Spearman’s correlation coefficient (*r*) with statistical significance evaluated based on a 2-tailed *P* value. A mixed-effects model with Šídák's multiple comparisons test was used to evaluate changes in K_max_ and CCt_min_ between baseline and the second visit, and to test differences in hormone distributions. A Kruskal-Wallis test with Dunn’s multiple comparisons test was used to evaluate the percent relative change in hormone levels from pre- to post-CXL. Missing data were excluded from further analyses (1 control eye was missing TKT at baseline, 2 control eyes were missing K_max_ and CCt_min_ values at baseline, 5 subjects were missing hormone levels at baseline, and 1 subject was missing hormone levels post-CXL). Data are presented as median, 95% confidence interval (CI), unless specified otherwise. Statistical significance was based on a *P* value of < 0.05.

## Results

In this study, the KC cohort was relatively young and predominately male with an average age of 25 years (Table 1). Patients with KC were clinically evaluated before CXL and then at a second visit ≥ 2 to 3 months after surgery for comparative analysis of KC severity, K_max_, and CCt_min_. The severity of KC before CXL was relatively high in this cohort, with a KC grade of ≥ 3 for 50% of the eyes undergoing CXL compared with 18% of the control eyes. A moderate number of patients had undergone CXL in the control eye (36%) ≥ 3 months before enrollment in this study, with only 2 patients having undergone corneal transplantation in the control eye.

**Table 1:** Clinical Characteristics of the KC Cohort

Characteristic	KC Cohort (n = 28)	
Age	25.0 ± 5.6 yrs	
Sex (male:female)	22:6 (79%:21%)	
Bilateral KC	21 (75%)	
KC severity (TKC) in CXL eye	1	1 (3%)
	1–2	3 (11%)
	2	7 (25%)
	2–3	3 (11%)
	3	9 (32%)
	3–4	5 (18%)
KC severity (TKC) in control eye	0	7 (25%)
	1	1 (3%)
	1–2	2 (7%)
	2	7 (25%)
	2–3	5 (19%)
	3	2 (7%)
	3–4	3 (11%)
	Missing	1 (3%)
Prior CXL in control eye	10 (36%)	
Prior corneal transplantation in control eye	2 (7%)	

CXL = corneal collagen crosslinking; KC = keratoconus; TKC = topographic keratoconus.

Continuous variables shown as mean ± standard deviation and categorical variables are shown as the number of patients (% of the cohort).

**Figure 1:**
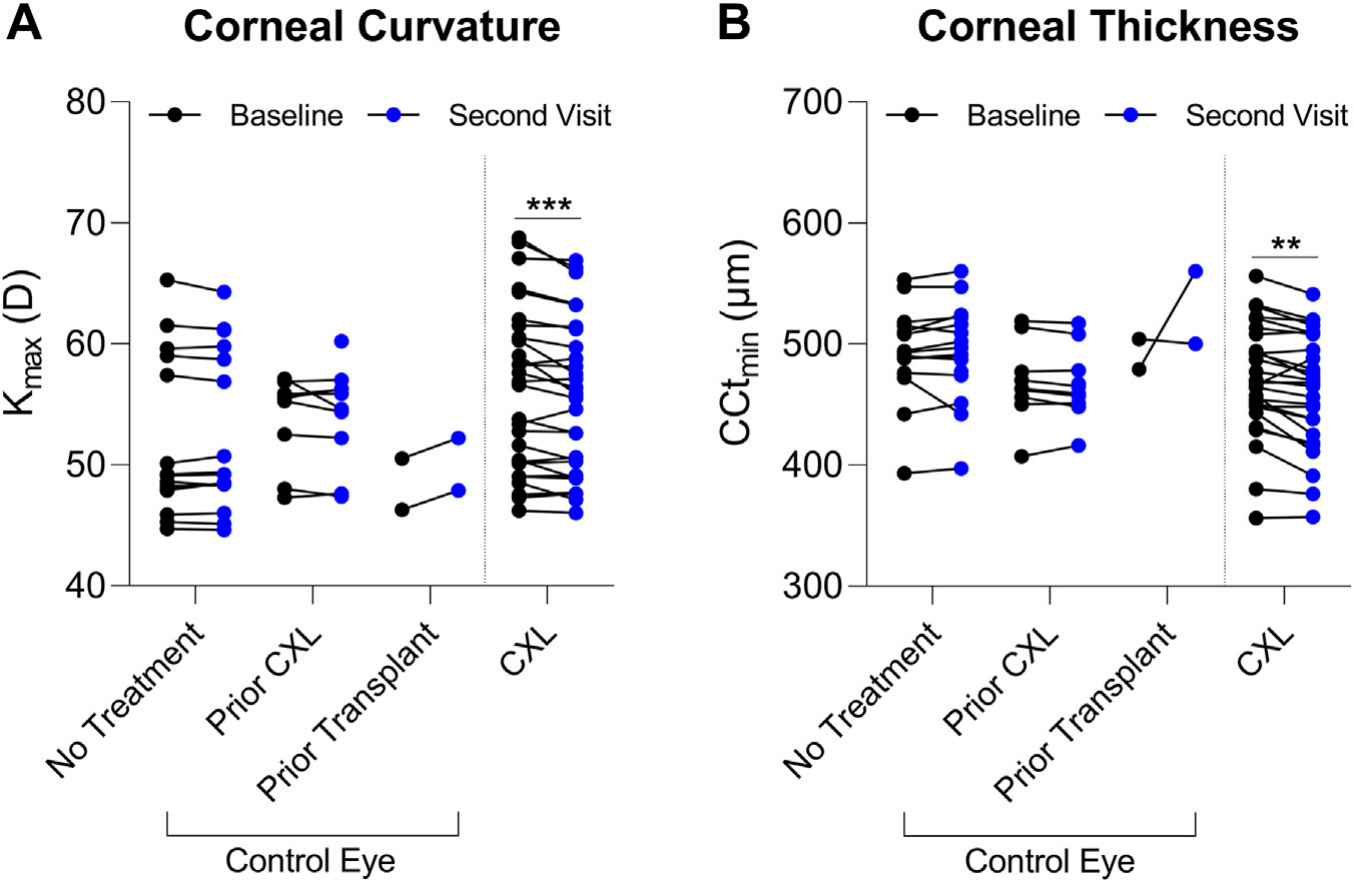
Changes in corneal curvature and thickness after corneal crosslinking (CXL). **A,** Comparison of maximum corneal curvature (K_max_) and (**B**) minimum central corneal thickness (CCt_min_) at baseline and at the second visit in the control eyes and CXL eyes from same subjects. Controls were stratified based on prior treatment, including CXL or corneal transplantation, that occurred between 3 and 33 months, or between 9 and 52 months, respectively, before the first study visit. Statistical significance based on a mixed-effects model with Šídák’s multiple comparisons test where ∗∗*P* ≤ 0.01 and ∗∗∗*P* ≤ 0.001.

Patients who underwent CXL showed a reduction by 2% in K_max_ from baseline (median, 56.8 D; 95% CI, 50.4–60.3) to the second visit (55.7 D; 95% CI, 50.4–58.8; *P* < 0.001; Fig 1A). The control group stratified by no treatment, prior CXL, and prior corneal transplantation had K_max_ values of 49.1 D (95% CI, 47.9–59.0), 55.5 D (95% CI, 48.0–56.9), and 48.4 D (95% CI, 46.3–50.5), respectively. Minimum central corneal thickness was reduced in CXL eyes by a median change of 10.5 μm (95% CI, 4.0–13.0; *P* = 0.002) by the second visit with no significant change in the collective control group from baseline (−1.0 μm; 95% CI, −4.0 to 4.0; Fig S2, available at www.ophthalmologyscience.org). Stratified based on prior treatments, baseline CCt_min_ values in the control group were 493.0 μm (95% CI, 476.0–515.0), 463.0 μm (95% CI, 450.0–514.0), and 491.5 μm (95% CI, 479.0–504.0) with no treatment, prior CXL, and prior corneal transplantation, respectively.

**Figure 3:**
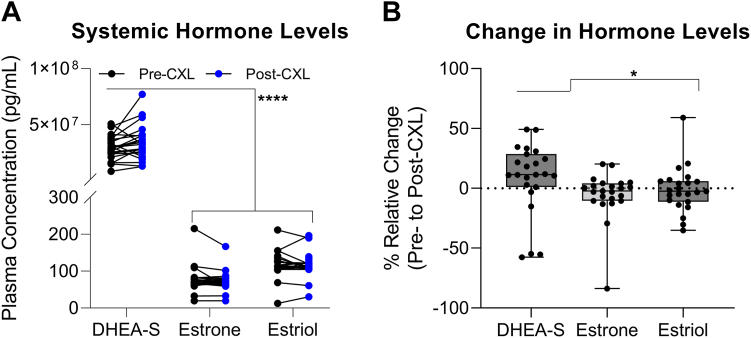
Systemic concentrations of dehydroepiandrosterone sulfate (DHEA-S), estrone, and estriol detected in plasma from patients with keratoconus before corneal crosslinking (CXL) and ≥ 2 to 3 months post-CXL. **A,** Patient-specific changes in systemic hormone levels are shown. Statistical significance was evaluated using a mixed-effects model with Šídák's multiple comparisons test where ∗*P* < 0.05. **B,** The relative % change in hormone levels at pre- and post-CXL for each patient, given that % relative change = (Δ [post-CXL vs. pre-CXL]/pre-CLX] × 100). Statistical significance was evaluated using a Kruskal-Wallis test with Dunn’s multiple comparisons test where ∗*P* < 0.05.

To assess systemic levels of hormones in the KC cohort, we measured plasma concentrations of DHEA-S, estrone, and estriol before and after CXL. Consistent with the literature,^23^ baseline systemic DHEA-S levels were significantly higher than estrone and estriol levels in circulation (DHEA-S, 3.0 × 10^7^ pg/ml; 95% CI, 2.5 × 10^7^ to 3.4 × 10^7^) versus estrone (72.2 pg/ml; 95% CI, 66.7–77.7) versus estriol (114.8 pg/ml; 95% CI, 106.0–125.3; *P* < 0.001; Fig 3A). After CXL, DHEA-S levels increased by 13% (95% CI, 3%–33%) from pre- to post-CXL and were significantly higher than the change in levels of estrone (−2%; 95% CI, −8% to 2%; *P* = 0.01) and estriol (−2%; 95% CI, −9% to 6%; *P* = 0.02; Fig 3B). In terms of patient-specific changes, estrogen and estriol levels decreased in most patients post-CXL (52% [12/23] and 56% [13/23], respectively), compared with only 22% (5/23) of patients showing a reduction in DHEA-S. Moreover, baseline DHEA-S levels were moderately lower in women (1.5 μg/ml; 95% CI, 0.8–2.6) than in men (3.0 μg/ml; 95% CI, 2.5–3.8; *P* = 0.03), and no sex-associated differences were associated with estrone and estriol levels, although these results are likely confounded by the small sample size (n = 4 females; Fig S4, available at www.ophthalmologyscience.org).

**Figure 5:**
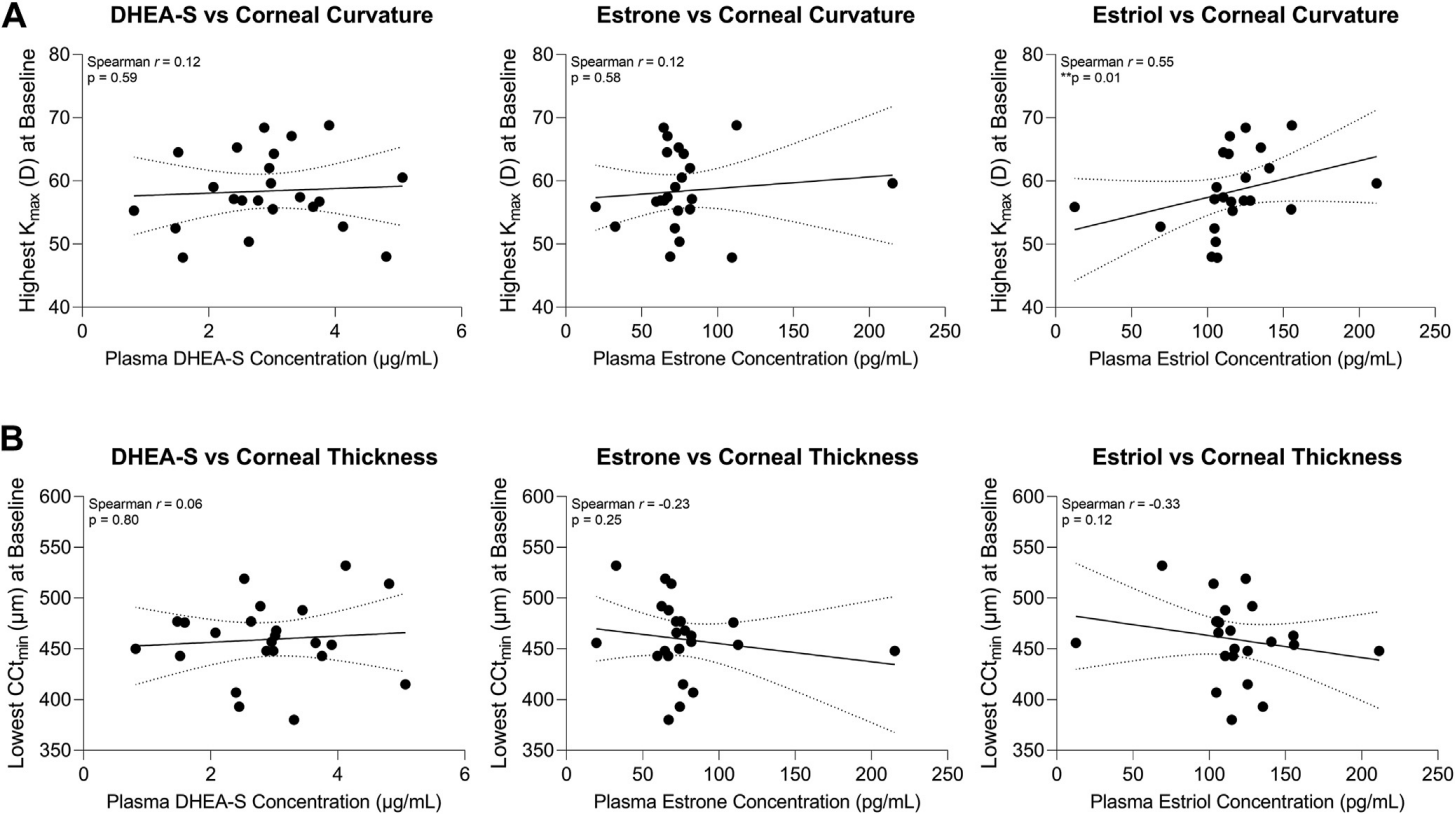
Investigation of potential associations between plasma hormone levels and clinical characteristics. Linear regressions of dehydroepiandrosterone sulfate (DHEA-S), estrone, and estriol with the (**A**) highest maximum corneal curvature (K_max_) and the (**B**) minimum central corneal thickness (CCt_min_) measured in each subject are shown (solid line) with 95% confidence intervals (dotted lines). Statistical significance was based on a Spearman correlation and a 2-tailed *P* value with ∗*P* < 0.05. D = diopter.

In terms of potential associations between circulating hormone levels and corneal parameters, baseline DHEA-S and estrone distributions showed little correlation with the highest K_max_ or lowest CCt_min_ for each subject, whereas estriol levels showed a significant positive correlation with the highest K_max_ detected at baseline (Spearman’s *r* = 0.55, *P* = 0.01) and no significant correlation with CCt_min_ (Fig 5). There was no significant association between age and hormone distributions in this KC population (Fig S6, available at www.ophthalmologyscience.org).

**Figure 7:**
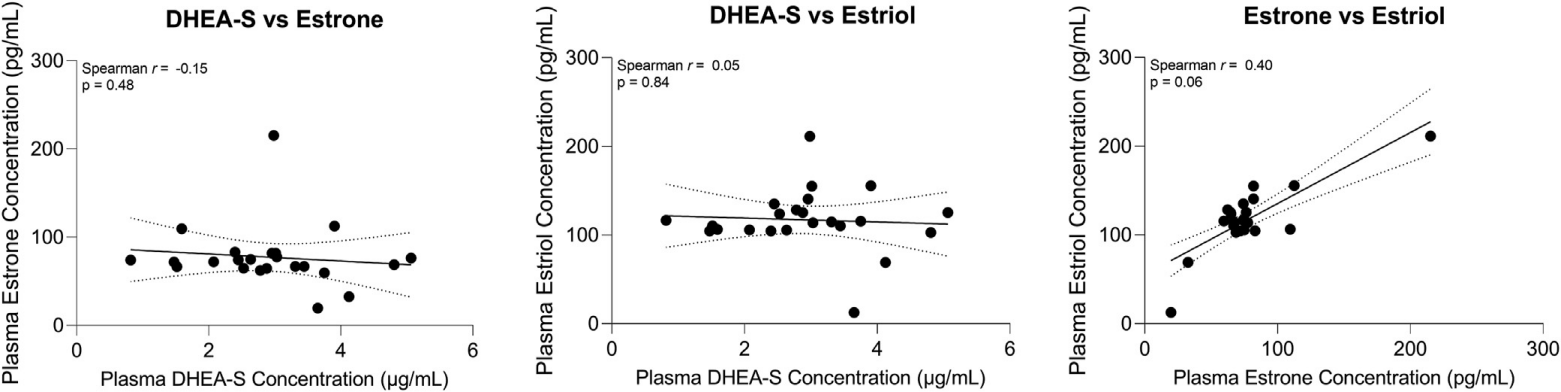
Associations between systemic hormone levels in keratoconus patients before corneal crosslinking. Linear regressions of dehydroepiandrosterone sulfate (DHEA-S), estrone, and estriol are shown (solid line) with 95% confidence intervals (dotted lines). Statistical significance was based on a Spearman correlation and a 2-tailed *P* value with ∗*P* < 0.05.

**Figure 8:**
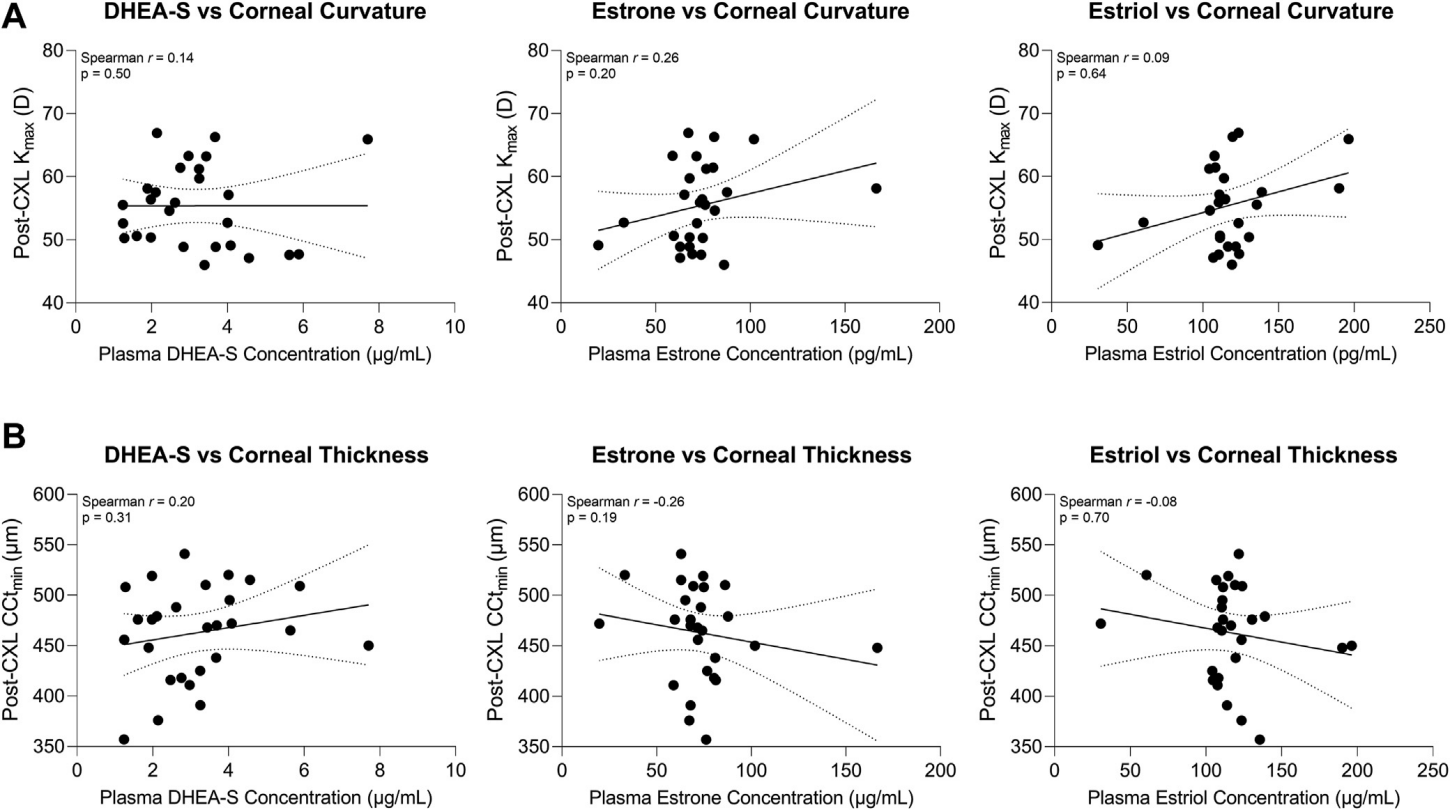
Investigation of correlations between systemic hormone levels and maximum corneal curvature (K_max_) and minimum central corneal thickness (CCt_min_) after corneal crosslinking (CXL). Linear regressions of dehydroepiandrosterone sulfate (DHEA-S), estrone, and estriol collected at the second visit with (**A**) K_max_ and (**B**) CCt_min_ in the CXL eye post-CXL are shown (solid line) with 95% confidence intervals (dotted lines). Statistical significance was evaluated using a Spearman correlation and a 2-tailed *P* value. D = diopter.

We next evaluated potential associations between the different hormones in circulation and observed a modest positive correlation between baseline estrone and estriol levels (Spearman *r* = 0.4, *P* = 0.06), though this finding did not reach our threshold for statistical significance (Fig 7). To determine whether hormone levels were correlated with the response to CXL based on corneal parameters, we evaluated the change in corneal curvature and thickness post-CXL relative to plasma levels of DHEA-S, estrone, and estriol detected at the same timepoint after CXL. Congruent with the pre-CXL findings, systemic DHEA-S and estrone levels showed no association with K_max_ values after CXL (Fig 8A). Interestingly, post-CXL circulating estriol levels were likewise not correlated with K_max_ in the CXL eye (Fig 8A) or the highest K_max_ value detected in either eye (Fig S9, available at www.ophthalmologyscience.org), although similar estriol levels were present at baseline (Fig 3). Moreover, post-CXL CCt_min_ was not associated with DHEA-S, estrone, or estriol plasma levels detected at the same timepoint (Fig 8B).

## Discussion

Consistent with prior studies,^24^^,^^25^ we found that CXL reduced maximum corneal curvature in CXL-treated eyes compared with baseline values. We observed a positive association between estriol levels and the highest maximum corneal curvature at baseline, with no strong correlation post-CXL, suggesting that the CXL intervention may overcome the systemic hormonal contributions in reducing KC progression. The association between higher estriol levels and increased corneal curvature at baseline is in agreement with case-control studies showing accelerated progression of KC with pregnancy,^19^^,^^26^ during which estriol levels are notably elevated.^27^ Moreover, although higher DHEA-S levels have been previously associated with KC,^16^^,^^17^^,^^28^ our findings revealed a slight increase in DHEA-S after CXL, in concert with reduced corneal curvature in the naive CXL eye, further supporting the idea that CXL may be effective independent of hormone distributions. In terms of cellular effects, CXL has been shown to reduce lactate production and modulate cellular bioenergetics in corneal fibroblasts derived from patients with KC^29^ accompanied by reduced expression of profibrotic markers, including α-smooth muscle actin and collagen type III,^30^ and denser collagen and proteoglycan deposition in vitro,^31^ supporting a mechanistic basis at the local site for the therapeutic benefit of CXL in reducing KC severity.

The effects of ultraviolet light exposure of the cornea on local and circulating hormones remain largely unknown. In an animal model, acute ultraviolet light exposure to the corneal surface has revealed the formation of estrogen adducts that have been attributed to promoting the sex-dimorphism observed in Fuch’s endothelial corneal dystrophy.^32^ In the skin, a higher degree of exposure to UV radiation has been associated with decreased circulating estradiol and estrone serum levels in postmenopausal women,^33^ in a process thought to be largely dependent on vitamin D synthesis and its influence on androgen and estrogen production in adipose tissue and the gonads.^34^^,^^35^

Androgen, estrogen, and progesterone receptors are highly expressed in the human cornea^36^^,^^37^ and have been posited to play potential roles in dry eye disease and autoimmune conditions with ocular manifestations.^38^, ^39^, ^40^ In general, estrogens are known modulators of biomechanical properties of various tissues,^41^^,^^42^ and estradiol treatment of the eye has been associated with leading to increased corneal stiffness and moderate myopic shifts in animal models.^43^^,^^44^ Our previous work has shown that the cornea is an extragonadal tissue that expresses luteinizing hormone and follicle-stimulating hormone.^15^^,^^45^ Human corneal fibroblasts derived from patients with KC have also been found to express differential levels of androgen, estrogen, and progesterone receptors; these differences are accompanied by altered responsiveness to hormone stimulation compared with controls.^46^^,^^47^ These findings suggest that systemic hormones, particularly estriol and estradiol, may influence corneal structure during KC.

Our current observational study provides supporting evidence that higher estriol levels are associated with increased corneal curvature pre-CXL in a predominately male KC population, suggesting that hormone status may be an important measure for consideration in terms of KC status. There are notable limitations in our study that may influence the generalizability of our findings, including the relatively small sample size, higher proportion of males to females enrolled, limited age-range of subjects, and the narrow distribution of hormones evaluated. Of note, given that KC is commonly a bilateral condition, prior treatments of the comparative control eye, including CXL and corneal transplantation, may have introduced important confounders in the interpretation of our data. Other confounders that may influence hormone distributions that were not controlled in our study include temporal contributions associated with the circadian rhythm and menstrual cycle. However, we posit that our findings are hypothesis-generating and may aid in the design of larger, more comprehensive studies to evaluate the potential role of systemic hormones in KC onset, progression, and response to treatment.
