# Supplementary material for: Prospective Observational Study Evaluating Systemic Hormones and Corneal Crosslinking Effects in Keratoconus
Source: Ophthalmol Sci. 2023 Jul 11;4(2):100364. doi: 10.1016/j.xops.2023.100364 (PMC10585634; doi:10.1016/j.xops.2023.100364)
Supplement: Supplemental figs [file mmc1.docx]

**Supplemental Materials for**

Prospective Observational Study Evaluating Systemic Hormones and Corneal Crosslinking Effects in Keratoconus

Lyly Van^1^, Sashia Bennett^2^, Sarah E. Nicholas^3,4^, Jesper Hjortdal^2^, Tina B. McKay^5,*^, Dimitrios Karamichos^3,4,6,*^

**Contents:**

**Supplemental Figure S2.** Changes in corneal curvature and thickness at 2 – 3 months following corneal crosslinking (CXL) in the collective control group versus the CXL group.

**Supplemental Figure S4.** Associations between biological sex and plasma DHEA-S, estrone, and estriol concentrations in KC patients before-and-after corneal crosslinking (CXL).

**Supplemental Figure S6.** Investigation of correlations between hormones (DHEA-S, estrone, and estriol) and age of the subject.

**Supplemental Figure S9.** Investigation of potential associations between plasma hormone levels and corneal curvature.


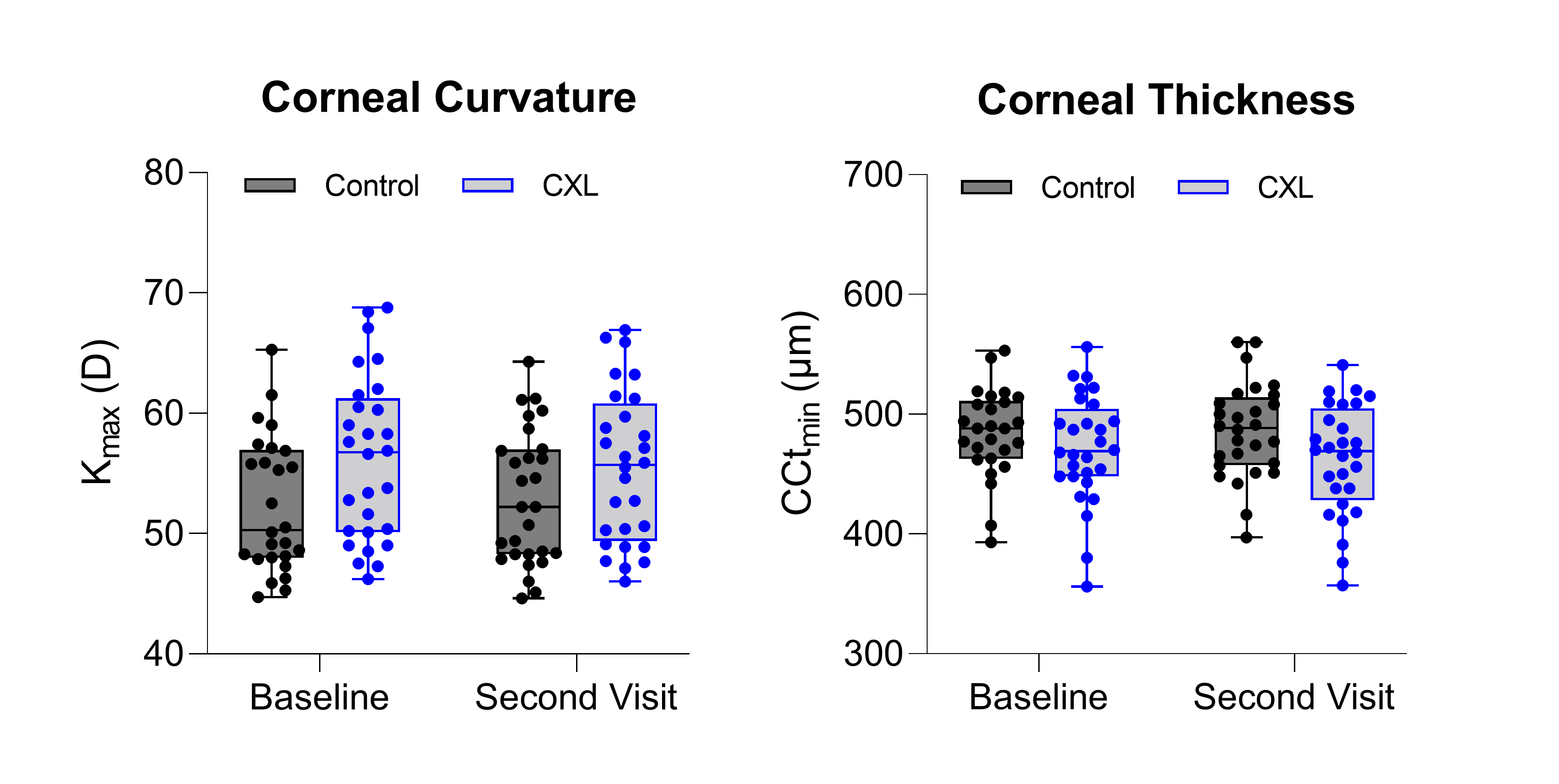


**Supplemental Figure S2.** Changes in corneal curvature and thickness following corneal crosslinking (CXL) in the collective control group versus the CXL group.


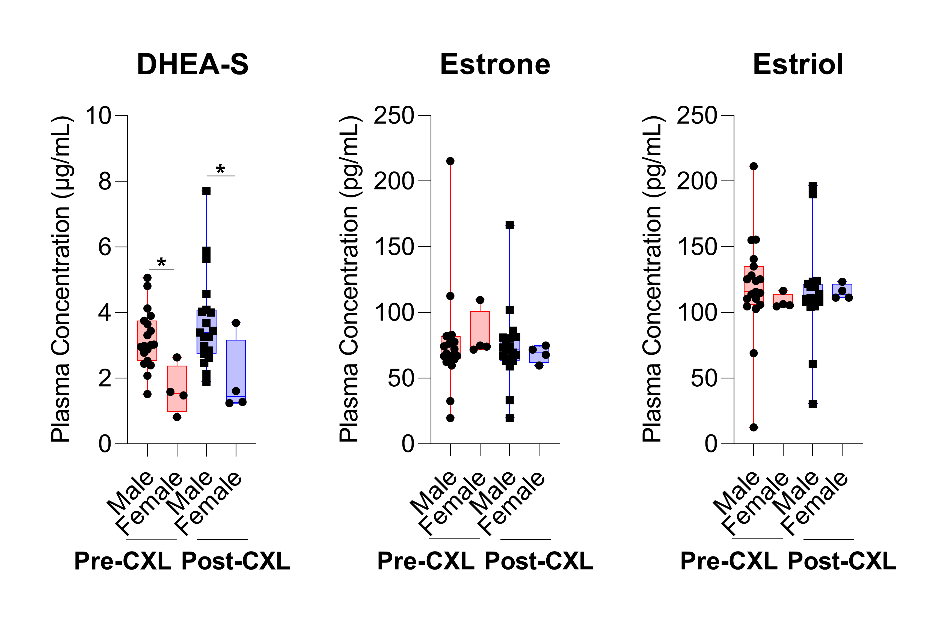
**Supplemental Figure S4.** Associations between biological sex and plasma DHEA-S, estrone, and estriol concentrations in KC patients before-and-after corneal crosslinking (CXL). Statistical significance based on a Kruskal-Wallis test with Dunn’s multiple comparisons test with *p < 0.05.


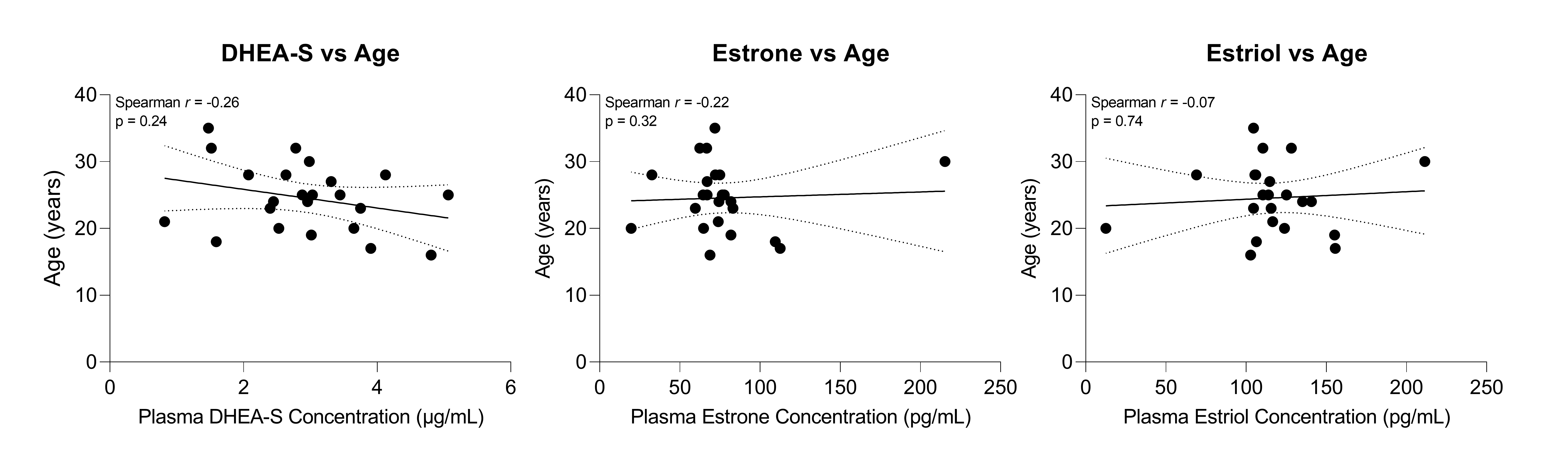


**Supplemental Figure S6.** Investigation of correlations between hormones (DHEA-S, estrone, and estriol) and age of the subject.


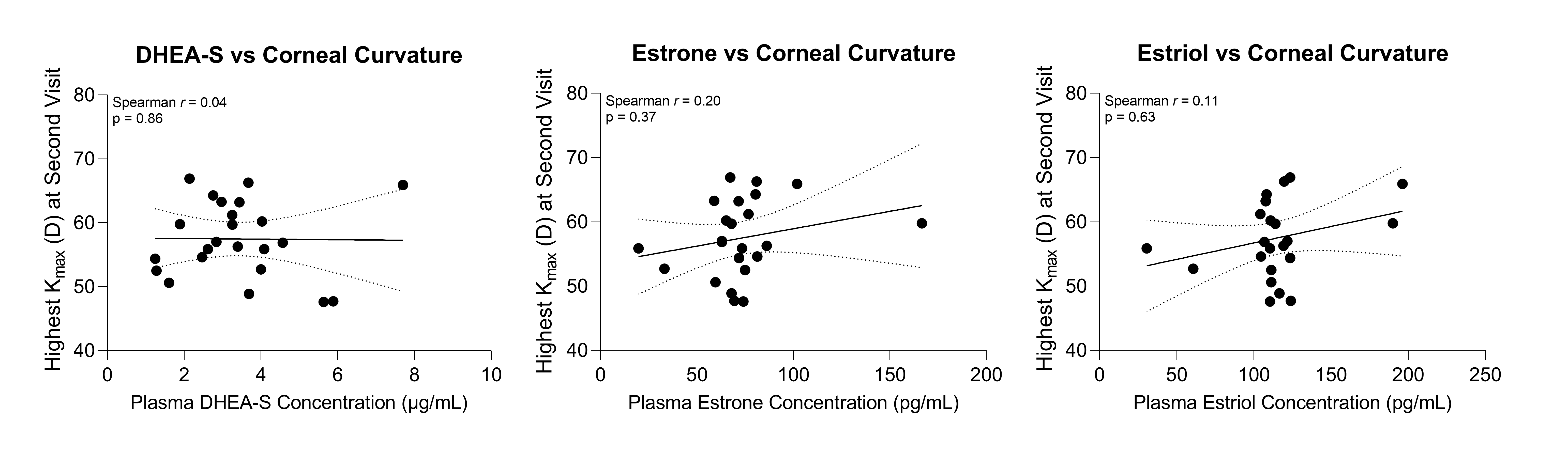


**Supplemental Figure S9.** Investigation of potential associations between plasma hormone levels and corneal curvature. Linear regressions of DHEA-S, estrone, and estriol with the highest maximum corneal curvature (K_max_) detected at the second visit. Statistical significance evaluated using Spearman correlations with a two-tailed p-value.
